# Post-Intensive Care Syndrome in Patients Suffering From Acute Subarachnoid Hemorrhage: Results From an Outpatient Post-ICU Aftercare Clinic

**DOI:** 10.7759/cureus.36739

**Published:** 2023-03-27

**Authors:** Dharmanand Ramnarain, Brenda Den Oudsten, Annemarie Oldenbeuving, Sjaak Pouwels, Jolanda De Vries

**Affiliations:** 1 Department of Intensive Care Medicine, Elisabeth-TweeSteden Ziekenhuis (ETZ), Tilburg, NLD; 2 Department of Medical and Clinical Psychology, Center of Research on Psychological and Somatic Disease (CoRPS), Tilburg University, Tilburg, NLD; 3 Department of Intensive Care Medicine, Saxenburgh Medisch Centrum, Hardenberg, Hardenberg, NLD; 4 Department of Intensive Care Unit, Elisabeth-TweeSteden Ziekenhuis (ETZ), Tilburg, NLD; 5 Department of General and Abdominal Surgery, Helios Klinikum, Krefeld, DEU; 6 Department of Psychology, Admiraal De Ruyter Hospital, Goes, NLD

**Keywords:** acute subarachnoid hemorrhage, depression, anxiety, ptsd, cognitive dysfunction, asah, post-intensive care syndrome, pics

## Abstract

Introduction

Survivors of an acute subarachnoid hemorrhage (aSAH) may suffer from a long-term neurological disability, cognitive impairment, anxiety, and depression, which can also be related to post-intensive care syndrome (PICS). The aim of this study was to examine the prevalence of PICS symptoms in post-intensive care (ICU) aftercare aSAH patients.

Methods

We conducted an observational cohort study in aSAH patients from a post-ICU aftercare clinic (ICU-AC). PICS symptoms were evaluated using the Impact of Event Scale-Revised (IES-R), Hospital Anxiety and Depression Scale (HADS), and a medical questionnaire for physical and cognitive functioning.

Results

A total of 110 patients were included. The prevalence of anxiety and depressive symptoms was 23.6% and 19.1%, respectively. Post-traumatic stress disorder (PTSD) was seen in 26.4%. Cognitive complaints were lack of concentration (63.6%), short-term memory loss (45.8%), and reduced speed of thinking (60.9%). The most reported physical complaints were fatigue (73.6%), limitations in daily activity (72.7%), muscle weakness (41.8%), pain (36.4%), and weight loss (30.9%). PICS symptoms related to all three domains were present in 30% of patients.

Conclusion

The prevalence of PICS in patients after aSAH is high. Even in patients without aSAH-related neurological impairment who were discharged home, a high prevalence of PICS symptoms was reported. Early screening for PICS should comprise all three domains and is important to facilitate a better tailored rehabilitation of these patients.

## Introduction

Acute subarachnoid hemorrhage (aSAH) from a ruptured intracranial aneurysm is a devastating disease associated with high mortality. Unruptured aneurysms are present in about 3% of the population [1]. The overall incidence of aSAH is nine per 100,000 person-years [2], and about 30% of the patients die within 24 hours [3,4]. The reported case fatality rate is 31% [5]. Most survivors have long-term disability or cognitive impairment and psychological symptoms, such as anxiety, depression, and limited daily functioning [3,6,7]. Even patients with good clinical outcomes after aSAH (patients with mild or no neurological symptoms due to the aSAH) can suffer from these symptoms [8-11] and can have a poor quality of life [12-14].

In 2010, the definition of post-intensive care syndrome (PICS) was introduced to describe the presence of new or worsened impairment in physical health, mental health (i.e., anxiety, depression, and post-traumatic stress disorder (PTSD)), and cognition after treatment in an intensive care unit (ICU) [15]. PICS is considered a multidimensional concept, with no clear diagnostic criteria.

There is an overlap of symptoms in aSAH patients surviving hospital care and the symptoms of PICS described above. Because nontraumatic brain injury can result in psychological and cognitive dysfunction, these symptoms are difficult to distinguish between temporary and reversible psychological and cognitive dysfunction occurring after a period of critical illness. That is the main reason why neurological patients are often excluded in outcome studies examining the effect on psychological and cognitive function. A neurological condition is often regarded as a confounding factor. For instance, in studies examining the effect of pharmacological as well as non-pharmacological interventions to treat delirium, neurological patients are excluded because delirium in these patients is rather difficult to distinguish between symptoms related to initial brain injury [16-18].

Studies investigating PICS in general ICU populations show different incidences of PICS symptoms [5,13-15]. To date, there are no reports of PICS incidence in patients surviving aSAH. Still, some have reported poor quality of life even five years after aSAH in patients who made a good recovery according to the Glasgow Outcome Scale [19-23]. These findings may suggest that besides already complex SAH-induced brain injury, other factors could contribute to one or more components of PICS. It remains challenging to distinguish PICS symptoms from symptoms related to SAH-induced brain injury.

There are no publications of studies reporting the full scale of PICS in patients surviving aSAH. The aim of this prospective observational cohort study was to examine the prevalence of PICS and search for ICU- and patient-related associated factors that could contribute to PICS in patients after aSAH.

## Materials and methods

Study design

This study was a prospective observational study of patients treated at the outpatient ICU aftercare clinic (ICU-AC) of Elisabeth-TweeSteden Ziekenhuis (ETZ) in Tilburg, the Netherlands. This hospital is a large nonacademic tertiary referral hospital for trauma, neurotrauma, and nontraumatic brain injury. The study was approved by the local Medical Ethics Committee (METC) (number: NW2017-23) and adheres to the principles laid down in the 1964 Declaration of Helsinki. It is conducted according to the Strengthening the Reporting of Observational Studies in Epidemiology (STROBE) statement [24].

Participants and procedure

Patients who visited the aftercare clinic between January 2012 and January 2017 who were treated for aSAH and received ICU care for at least 24 hours and who were 18 years or older at ICU admission were included. Four weeks prior to the visit to the outpatient ICU-AC, patients were sent different postal questionnaires screening for anxiety and depression, PTSD, physical complaints, and subjective cognitive dysfunction. Patients were asked to complete the questionnaires one week before visiting the outpatient ICU-AC. These questionnaires were analyzed by an intensivist and a specialized ICU nurse. They also saw all the patients. Any misunderstanding of certain questions of the questionnaires was clarified if needed and corrected. Patients who were unable to visit the outpatient ICU-AC because of clinical rehabilitation or poor health were invited to visit our clinic at a later stage if possible. In case of treatable impairment in physical, psychological, or cognitive domains, patients were referred to their treating medical specialists or general practitioner (GP). In the Netherlands, the GP is the primary healthcare professional for outpatients. They are responsible for treating and coordinating the care of outpatients. If there is a need for additional specific care, (s)he is able to refer the patient to specific healthcare professionals or institutions.

Data collection

Clinical and demographic data were retrieved from the electronic patient records. Demographic data consisted of age and gender. Medical data included initial diagnosis, type of admission (i.e., referring specialism, emergency surgical, and referral from another hospital), length of stay (LOS) in the hospital and/or ICU, the Acute Physiology and Chronic Health Evaluation II and IV (APACHE II and APACHE IV), organ support in the ICU (i.e., mechanical ventilation and hemodialysis), and presence of delirium at any time. Data regarding complications of aSAH, such as hydrocephalus, rebleed, vasospasm and meningitis, and secondary ischemia, were recorded, as well as data on drugs administered during ICU treatment, including sleep medication, other benzodiazepines, antipsychotics, inotropes and vasopressors, corticosteroids, and opiates.

PICS definition

We defined PICS as having complaints in all three domains: physical, cognitive, and psychological (i.e., anxiety, depression, and PTSD) functioning.

Physical Functioning

An in-hospital-developed questionnaire based on the research performed by Griffiths et al. [25] and Jones et al. [26] was used to evaluate physical health symptoms. This questionnaire consists of 16 questions regarding frequently described physical complaints of post-ICU patients [15,25,26]. Patients were asked if they experienced physical complaints such as fatigue, muscle weakness, pain or any limitation in daily functioning, weight loss, or loss of hearing or sight. Patients could answer “yes” or “no” and were free to comment more if they wanted to.

Subjective Cognitive Functioning

Three specific questions concerning cognitive functioning were asked. These questions assessed the presence of short-term memory deficits, problems with concentration/attention, and slow speed of thinking. For each question, the patient responded “yes” or “no.” We defined the presence of cognitive dysfunction when at least one of the three questions was answered “yes.”

Psychological Functioning

The 14-item Hospital Anxiety and Depression Scale (HADS) assesses symptoms of anxiety and depression [27]. The HADS consists of two subscales: anxiety (HADS-A) and depression (HADS-D). The response scale ranges from 0 to 3, resulting in a total score ranging from 0 to 21 for each subscale. A subscale score of 8 or above indicates a possible anxiety or depression disorder [28]. This is the most commonly used and validated tool in post-ICU patients.

Symptoms of PTSD were assessed using the Impact of Event Scale-Revised (IES-R) questionnaire [29]. This questionnaire consists of 22 statements with a 5-point scale (0-4). The total score ranges from 0 to 88. A cumulative score of 24 or higher indicates the possible presence of PTSD, and a score of 33 or higher indicates a strong possible presence of PTSD [30-32].

Statistical analysis

We used descriptive statistics to describe the prevalence of demographic data (age, sex, and disease severity scores (APACHE II and APACHE IV)), aSAH-related characteristics (Fisher’s score, Glasgow Coma Scale (GCS) score on admission, World Federation of Neurosurgical Societies (WFNS) score, Hunt and Hess grade, and aneurysm location), and ICU-related factors, such as ICU delirium, mechanical ventilation, renal support, sedative use, opioids, vasoactive medications, PICS symptoms, anxiety, depression, PTSD, cognitive functioning, and physical symptoms.

Continuous variables were described as mean ± standard deviation; categorical variables were described as frequency (percentage). To study the relationship between continuous variables age, ICU LOS, hospital LOS, APACHE II and APACHE IV, mechanical ventilation duration, sedative use, opiates, vasoactive medications, and PICS symptoms, the Mann-Whitney U test was used. To study the association between aSAH-related characteristics (aneurysm location, GCS, WFNS, Fisher’s score, Hunt and Hess grade on admission, complications, and ICU delirium), chi-square tests were used. Missing data on multi-item questionnaires were analyzed; for missing data on item level, we used mean item imputation. Database preparation and all statistical analyses were conducted using Statistical Package for the Social Sciences (SPSS) version 25 (IBM SPSS Statistics, Armonk, NY, USA). A p-value of <0.05 was considered statistically significant.

## Results

**Figure 1:**
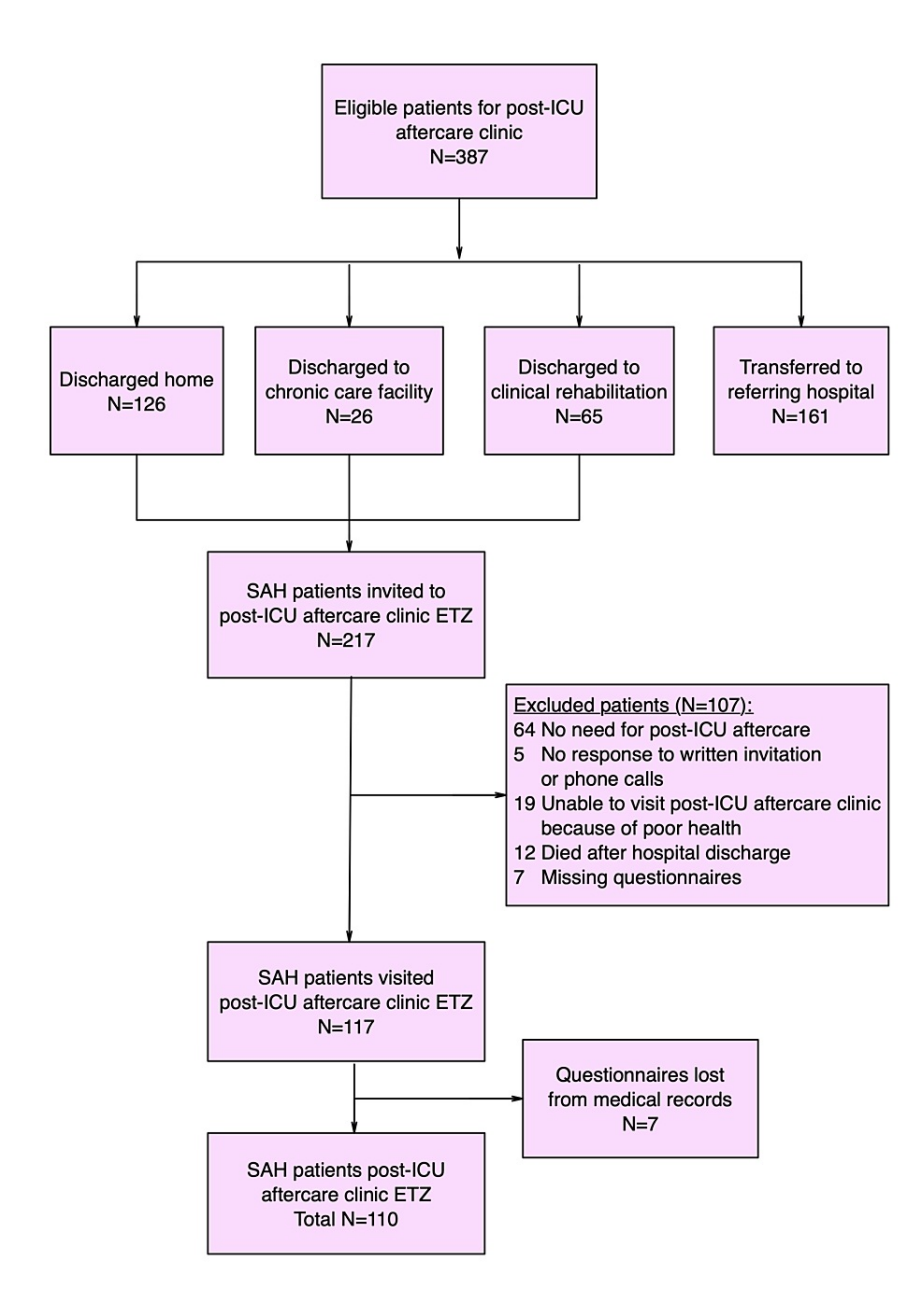
Flowchart of the selection of cohort of SAH patients who visited the post-ICU aftercare clinic. SAH: subarachnoid hemorrhage, ICU: intensive care unit, ETZ: Elisabeth-TweeSteden Ziekenhuis Hospital

A total of 378 eligible SAH patients were treated in the ICU and transferred to the hospital ward for further recovery. Of these patients, 126 were discharged home because of assumed good functional recovery, meaning that they could in general perform activities of daily living independently, 26 patients were discharged to chronic care facilities due to inability to be cared for in their home situation, 65 patients were transferred to a clinical rehabilitation center, and 161 patients were transferred to referring hospitals near their own home addresses. Their own neurologist at that hospital then coordinated further recovery. As a consequence, these patients were not invited to our outpatient ICU-AC. Two hundred seventeen patients were invited to visit the ICU-AC. Because of various reasons, 117 visited our ICU-AC. Seven patients were excluded from analyses because of missing data in the questionnaires, resulting in 110 patients who visited the outpatient ICU-AC (Figure 1).

In Table 1, the demographic and clinical characteristics of the patients are described.

**Table 1:** Baseline demographics and ICU characteristics. SAH: subarachnoid hemorrhage, LOS: length of stay, APACHE: Acute Physiology and Chronic Health Evaluation, AVM: arteriovenous malformation, ICU: intensive care unit, WFNS: World Federation of Neurosurgical Societies

SAH patient characteristics	Patients (N=110)
Age (years), median (range)	60 (31-81)
Sex (male), number (%)	35 (31.8%)
APACHE II, median (range)	11 (3-34)
APACHE IV, median (range)	36 (10-115)
ICU LOS (days), median (IQR)	6 (5-11)
Hospital LOS (days), median (IQR)	15 (12-21)
Mechanical ventilation (yes), number (%)	35 (31.8%)
Days on mechanical ventilation, median (IQR)	4 (2-9)
Renal support (yes), number (%)	0 (0%)
ICU delirium (yes), number (%)	27 (24.5%)
ICU discharge to post-ICU aftercare clinic (days), median (IQR)	135 (107-161)
Hospital discharge to post-ICU aftercare clinic (days), median (IQR)	127 (99-155)
Complications of SAH, number (%)	
Hydrocephalus	35 (31.8%)
Rebleed	4 (3.6%)
Vasospasm	19 (17.5%)
Secondary ischemia	5 (4.5%)
Secondary meningitis	3 (2.7%)
WFNS score, number (%)	
1	55 (50%)
2	20 (18.2%)
3	4 (3.6%)
4	17 (15.5%)
5	14 (12.7%)
Source of SAH, number (%)	
No aneurysm	14 (12.7%)
Arterial aneurysm	94 (85.4%)
“Other” (perimesencephalic, AVM)	2 (1.8%)
Aneurysm location, number (%)	
Anterior circulation	46 (41.8%)
Middle circulation	20 (18.2%)
Posterior circulation	28 (25.5%)
Fisher’s score on admission	
1	8 (7.3%)
2	10 (9.1%)
3	17 (15.5%)
4	63 (57.3%)
Hunt and Hess grade on admission	
1	55 (50%)
2	21 (19.1%)
3	3 (2.7%)
4	17 (15.5%)
5	14 (12.7%)

Prevalence of PICS symptoms

Missing data were found in up to 8.2% of the items of the IES-R questionnaires, 2.7% of the HADS, and 2.7% of items related to cognitive functioning. In the medical questionnaires, up to 3.6% missing values were found. Mean item imputation was performed on IESR-R and HADS-related items. No difference in domain-specific diagnosis was found after mean item imputation compared to complete case analysis. According to our definition, 33 (30%) patients had complaints in all three domains and thus had PICS. Of the other patients, 48 (43.7%) had complaints related to two domains, and only six (5.5%) patients had no complaints related to any of these domains (Table 2).

**Table 2:** Prevalence of PICS complaints according to their domains. PICS: post-intensive care syndrome

PICS domains	Patients (number)	Percentage (%)
No PICS symptoms	6	5.5
Physical only	23	20.9
Fatigue	81	75
Limitations in daily activity	80	74.1
Psychological only	1	0.9
Cognitive only	1	0.9
Physical + psychological	7	6.4
Physical + cognitive	41	37.3
Psychological + cognitive	0	0
All three domains	33	30

Demographic factors were not related to anxiety, depression, or PTSD. Of all mentioned ICU-related factors, only the duration of mechanical ventilation was associated with PTSD (χ2=7.159, p=0.028). In Table 3, all reported physical, psychological, and subjective cognitive complaints are summarized.

**Table 3:** Prevalence of self-reported somatic, cognitive, and psychological complaints. PTSD: post-traumatic stress disorder

Complaints	Number (%)
Somatic complaints	
Fatigue	81 (75)
Limitations in daily activity	80 (74.1)
Muscle weakness	46 (42.1)
Pain	40 (37.4)
Weight loss	34 (32.1)
Visual complaints	26 (24.1)
Hearing loss	21 (19.6)
Urinary complaints	21 (19.6)
Hair loss	20 (18.5)
Hoarseness	14 (13)
Skin problems	13 (12)
Dyspnea	12 (10.9)
Problems eating	6 (5.6)
Problems swallowing	3 (2.8)
Cognitive complaints	
Impaired concentration	70 (64.8)
Short-term memory loss	49 (45.8)
Reduced speed of thinking	40 (37.4)
Psychological symptoms	
PTSD	30 (27.3)
Anxiety	26 (23.6)
Depression	21 (19.1)

Physical complaints

Fatigue (n=81, 75%), limitations in daily activity (n=80, 74.1%), muscle weakness (n=46, 42.1%), pain (n=40, 37.4%), and weight loss (n=34, 32.1%) were the top five most reported physical complaints.

Subjective cognitive complaints

The reported cognitive complaints were problems with concentration (63.6%), short-term memory problems (45.8%), and reduced speed of thinking (60.9%).

PICS in good functional outcome aSAH versus poor functional outcome aSAH

Nineteen patients were discharged and went home because of good clinical recovery without SAH-related functional impairment. Ninety-one patients were discharged to chronic care facilities or clinical rehabilitation centers. In both groups, a high incidence of PICS was seen. PICS symptoms were not significantly different between both groups (Table 4).

**Table 4:** PICS in aSAH patients discharged home versus discharged to a chronic care facility or rehabilitation clinics. HADS: Hospital Anxiety and Depression Scale, aSAH: acute subarachnoid hemorrhage, CCF: chronic care facility, RC: rehabilitation clinic, ns: not significant, PTSD: post-traumatic stress disorder

aSAH patients	Discharged home (n=19)	Discharged to CCF or RC (n=91)	p-value
HADS anxiety score >8 (%)	31.6	22	ns
HADS depression score >8 (%)	31.6	16.5	ns
PTSD (%)	31.6	26.4	ns
Reduced speed of thinking (%)	15.8	40.7	0.046
Impaired concentration (%)	89.2	58.2	0.013
Short-term memory loss (%)	36.8	46.2	ns

Psychological symptoms

The prevalence of symptoms of anxiety and depression in SAH patients was 24.3% (n=26) and 19.6% (n=21), respectively. Additionally, 28.6% (n=30) of the aSAH patients had PTSD symptoms.

Complicated course of aSAH and PICS

Hydrocephalus and vasospasm were the two most seen complications of SAH in this cohort. All patients with complications had symptoms in all three domains of PICS. In Table 5, significant associations between hydrocephalus, vasospasm, rebleed, meningitis, and secondary ischemia and PICS symptoms are shown. The p-value was adjusted for multiple comparisons using the Bonferroni test. Although complaints were reported in all domains, no significant associations were found in patients with hydrocephalus (Table 6). Rebleed after aSAH was significantly associated with PTSD symptoms (χ2=4.392, p=0.0036), impaired vision (χ2=5.894, p=0.015), and impaired hearing (χ2=8.077, p=0.004). Secondary meningitis was significantly associated with muscle weakness (χ2=4.159, p=0.041) and weight loss (χ2=6.538, p=0.011). Vasospasm during ICU treatment was significantly associated with impaired vision (χ2=4.904, p=0.027). No significant association was seen between secondary ischemia and any of the physical, cognitive, and psychological symptoms of PICS. ICU delirium was associated with problems with eating (χ2=6.305, p=0.012) and short-term memory loss (χ2=4.160 p=0.0041).

**Table 5:** Complications between aSAH and ICU delirium and PICS symptoms. †Adjusted for multiple testing (Bonferroni method) ‡No significant associations with physical, cognitive, and psychological symptoms aSAH: acute subarachnoid hemorrhage, ICU: intensive care unit, PICS: post-intensive care syndrome, PTSD: post-traumatic stress disorder

Complications of SAH	PICS symptoms	Chi-square	p-value†
Rebleed	Impaired vision	5.894	0.015
	Impaired hearing	8.077	0.004
	PTSD	4.392	0.036
Vasospasm	Impaired vision	4.904	0.027
Secondary meningitis	Muscle weakness	4.159	0.046
	Problems with eating	4.538	0.033
	Weight loss	6.538	0.011
Hydrocephalus	‡	‡	‡
Secondary ischemia	‡	‡	‡
ICU delirium	Problems with eating	6.305	0.012
	Short-term memory loss	4.160	0.041

**Table 6:** PICS symptoms and complicated course in aSAH patients. PICS: post-intensive care syndrome, aSAH: acute subarachnoid hemorrhage, PTSD: post-traumatic stress disorder, +: positive association, -: no association, +^‡^: significant association with an adjusted p-value for multiple testing (Bonferroni method)

PICS symptoms	Hydrocephalus	Rebleed	Vasospasm	Secondary meningitis	Secondary ischemia	ICU delirium
Physical complaints						
Fatigue	+	+	+	+	+	+
Limitations in daily activity	+	+	+	+	+	+
Muscle weakness	+	+	+	+^‡^	+	+
Pain	+	+	+	+	+	+
Weight loss	+	+	+	+^‡^	+	+
Visual complaints	+	+^‡^	+^‡^	+	+	+
Hearing loss	+	+^‡^	+	+	-	+
Urinary complaints	+	+	+	-	-	+
Hair loss	+	+	+	-	-	+
Hoarseness	+	+	+	-	-	+
Skin problems	+	+	+	+	-	+
Dyspnea	+	+	+	-	-	+
Problems eating	+	-	+	+^‡^	-	+^‡^
Problems swallowing	+	-	+	-	-	+
Cognitive complaints						
Impaired concentration	+	+	+	+	+	+
Short-term memory loss	+	+	+	+	+	+^‡^
Reduced speed of thinking	+	+	+	+	+	+
Psychological symptoms						
PTSD	+	+^‡^	+	-	+	+
Anxiety	+	+	+	+	+	+
Depression	+	+	+	-	+	+

## Discussion

To our knowledge, this is the first study evaluating symptoms comprising all domains of PICS in patients suffering from acute SAH. This study shows that aSAH patients can have PICS and thus suffer from psychological, cognitive, and physical complaints. This is true for patients with and without a good functional outcome. Unrecognized PICS symptoms can become chronic when untreated and can hamper long-term improvement and quality of life.

In this prospective observational cohort study, we examined the physical, cognitive, and psychological domains of PICS. Most studies are limited to only one domain addressing the psychological or cognitive domain of PICS without mentioning the complete evaluation of PICS symptoms in these patients.

We found a high prevalence of PICS symptoms in aSAH patients with complaints in all three domains at 33%. In our cohort, only six (5.5%) patients had no complaints related to PICS. With a median of four months after being discharged from the ICU, 24.3% had anxiety, 19.6% had depression, and 11.4% had probable PTSD. In a recent systematic review, 0%-61.7% of patients had depression after SAH, with a weighted average prevalence of 28.1% [6]. An explanation for this difference could be the use of different screening tools used to measure depression and measurement at different time points after the initial SAH. Also, in this study, relatively good outcomes in SAH patients were studied, which could explain the lower prevalence of depression.

Cognitive dysfunction, anxiety, depression, and PTSD, and some physical symptoms such as fatigue are associated with diminished short-term and long-term quality of life in these patients [9,33,34].

As stated earlier, PICS is a multidimensional concept and not yet fully understood. In a recent meta-analysis, 60 risk factors for PICS in general ICU patients have been described when considering each domain separately [35]. The incidence of PICS in general ICU survivors is reported to be around 50%-70%, and the syndrome can persist even 5-15 years after ICU treatment [36]. The true incidence of PICS in patients suffering from aSAH is not known, but anxiety, depression, and cognitive dysfunction are reported to be around 50%-60% in these patients [37]. The reported prevalence of physical symptoms such as fatigue varies from 31%-90% [8].

Our prevalence of physical complaints was high and comparable with that reported in general post-ICU patients [38]. Physical complaints were reported in many patients with the top five complaints being fatigue (73.6%), limited exercise tolerance leading to limitations in daily activity (72.7%), muscle weakness (41.8%), pain (36.4 %), and weight loss (30.9%). There are few studies reporting physical complaints after SAH. Huenges Wajer et al. [39] evaluated complaints in 67 SAH patients during outpatient clinic visits. After six months, 64% experienced restrictions in housekeeping, chores in and around the house, and during physical exercise, whereas Dulhanty et al. [40] used written questionnaires to assess the need of SAH patients after 1-2 years and 3-5 years. The most commonly reported physical complaints were headaches and other pains at 58% and 45%, respectively. These two studies are in line with our findings showing that many patients who are considered as good outcome patients experience complaints in daily functioning. In our study, 74.1% of patients experienced limitations in daily functioning after 3-4 months. In a recent study, Custal et al. [41] reported long-term symptoms in 351 SAH patients with assumed good outcomes but having impairments in activities (24%), pain (33.4%), and anxiety/depression (42.5%) after a period of 12 months of follow-up.

The reported fatigue rates after SAH ranges from 31% to 90% [16]. Fatigue is a complex disorder that can be extremely debilitating [42,43]. Currently, the mechanism of fatigue after SAH is not known, and no reliable treatments are available. A recent meta-analysis found fatigue to be associated with cognitive dysfunction [44]. The authors concluded that the management of cognitive dysfunction may improve fatigue levels among SAH patients and that targeted therapy with less cognitive load could improve levels of fatigue in these patients [44].

Muscle weakness was seen in 41.8% of patients. Muscle weakness has been described in SAH patients showing reduced knee muscle strength of up to 28%-47% compared to healthy controls [45]. Pain was reported in 36.4% of patients. Pain has been associated with depression, cognitive dysfunction, poor quality of life, and increased consumption of pain medication [46,47]. In our cohort, pain was not related to any of the psychological and cognitive symptoms. Perhaps, the follow-up period in our study was too short to find any association. Also, the group size may have been too small to find this association. Nevertheless, it is clear that pain is often under-recognized and undertreated. When pain is recognized and treated, early prognosis regarding PICS symptoms could be improved. More research is needed to understand the pathophysiology of post-SAH-related pain.

Cognitive impairment was highly prevalent, with short-term memory problems, lack of concentration, and slow speed of thinking presented in 63.6%, 45.8%, and 60.9% of patients, respectively. Although cognition is complex and comprises multiple domains that can be screened for, we did not use elaborate questionnaires or specific tests to screen for cognitive dysfunction. Instead, we asked for three basic components of cognition, memory, attention, and speed of thinking, which are directly related to patients’ subjective cognitive functioning. Our findings are consistent with earlier studies reporting a range of 7%-73% in at least one aspect of cognitive function [10,37,48,49]. ICU delirium in our cohort was seen in 24.5% of SAH patients and was significantly associated with cognitive dysfunction (i.e., short-term memory loss). Delirium in SAH patients is a known complication occurring in about 24%-50% of patients [50-52]. In a study by Reznik et al. [52] on a cohort of 305 SAH patients, agitated delirium was seen in 50% of the patients. Delirium, in this study, was not associated with cognitive impairment after 12 months [52]. This could indicate that cognitive dysfunction in SAH patients may be reversible to some extent as we measured cognition early at three months after hospital discharge.

The prevalence of PTSD was 11.4%. Earlier reported prevalence rates in older studies vary between 6%, 19%, 32%, and 60% [53,54]. Hedlund et al. [55] reported a prevalence of 18% after seven months. There was no significant association between demographic factors and ICU-related factors, ICU delirium, steroid use, vasoactive or inotropic drugs, sedatives, and PTSD. In a prospective cohort of 143 neurology outpatient clinic SAH patients, PTSD was reported in 26% after three months [34]. In this study, no significant relationship between demographic and SAH-related factors was found. The only related factor for PTSD was the passive coping style of the patient. Unfortunately, we did not assess nor screen for this in our study. Also, ICU distress is a known factor; however, we did not assess ICU distress and its effects in the current study. All patients were managed according to pain, agitation, and delirium protocol [56].

Complications of SAH and PICS

Hydrocephalus and vasospasm were the two most seen complications in our cohort. All patients with complicated courses of SAH had multiple physical symptoms and had complaints in the cognitive and psychological domains. Patients with hydrocephalus showed multiple complaints, but no significant association was seen between hydrocephalus and any of the PICS symptoms. In a case series of 51 aSAH patients, hydrocephalus was associated with cognitive dysfunction [48]. Vasospasm was significantly associated with impaired vision. We have no clear explanation for this association. The pathogenesis of vasospasm is complex, and it is still not understood why some patients develop vasospasm. A combination of patient-related, genetic, and environmental factors may influence the development of vasospasm [57].

Good clinical outcome SAH patients and PICS

Patients with good clinical outcomes after aSAH who were discharged home showed a high prevalence of PICS. This was similar in patients with poor outcomes who were discharged to rehabilitation clinics. Although no statistically significant differences were found between these two groups and despite the fact that some patients who were discharged to clinical rehabilitation or chronic care facilities were still recovering during the ICU aftercare clinic visit, this was a surprising observation. We know of only two reports of PTSD in good outcome SAH patients that were published [53,54]. Apparently, patients with assumed good clinical outcomes who were discharged home still have significant hidden psychological symptoms, cognitive dysfunction, and physical complaints.

Also, after a period of rehabilitation, patients can still have symptoms related to PICS [58]. Therefore, screening for PICS symptoms in presumed good outcome SAH patients and in patients after rehabilitation is of great importance to improve outcomes in these patients.

Treatment and screening

If recognized, many psychological and physical complaints of PICS can be treated. For instance, various treatments can be performed by a psychologist for anxiety, depression, and PTSD, such as cognitive behavioral therapy, eye movement desensitization and reprocessing (EMDR), narrative exposure therapy, eclectic psychotherapy, supportive counseling, and acceptance and commitment therapy (ACT) [59,60]. Pain can be caused by SAH-related brain injury itself or due to problems in extremities or muscles or nerves. When pain is related to the stroke, amitriptyline, lamotrigine, and GABA receptor agonist are advised as first-line pharmacotherapeutic options [61]. Also, non-pharmacotherapeutic interventional therapies such as motor cortex stimulation or transcranial magnetic stimulation have been shown to provide relief in difficult-to-treat patients [62].

Early screening for PICS symptoms in all three domains can be beneficial for patients and may contribute to a better tailored rehabilitation. A multidisciplinary team approach for screening, in which more specialists combine their knowledge, for instance, a rehabilitation doctor, (neuro)psychologist, physiotherapist, neurologist, neurosurgeons, pain specialist, and intensivists, could be beneficial for these patients. Studies focusing on this multidisciplinary team approach are currently conducted in our hospital. Also, identifying possible ICU-related risk factors related to PICS could help prevent PICS in these patients. The ABCDEF bundle describing daily awakening, spontaneous breathing trials, coordination of care and communication with other disciplines, delirium prevention, early mobilization, and family participation in the ICU showed improvements in survival, delirium-free days, and coma-free days and better post-ICU conditions [63].

Strengths and limitations

This is the first study describing the full spectrum of PICS in a cohort of SAH patients. This study clearly shows that SAH patients can experience multiple limitations in physical, cognitive, and psychological functioning. Also, we made an attempt to better define PICS symptoms for future research and for a better understanding of the full spectrum of PICS. The cohort of aSAH patients can be considered as relatively good outcome patients without severe disability. Only patients who were able to visit the post-ICU aftercare and were able to fill in the questionnaires were included in this study. The reported results can therefore be biased (confounding and selection bias).

The diagnosis of anxiety, depression, PTSD, and cognitive dysfunction was based on questionnaires. Psychiatrists or psychologists did not assess patients, so an overestimation or underestimation is possible. All patients were not only screened with posted questionnaires but also were also seen in person by an intensivist and ICU nurse in our outpatient ICU-AC. False or misinterpreted questions or diagnoses could be corrected if necessary, during this contact with the patient. The reported results in this rather small cohort of patients could be an over- or underestimation of the prevalence of PICS. Larger studies on aSAH patients are needed to address the prevalence of PICS.

Future recommendation

Future studies focusing on outcomes in SAH patients should regard the full spectrum of PICS to distinguish between SAH-related brain damage and PICS-related symptoms. Also, the effect of early tailored treatment in aSAH patients should be addressed in larger studies. A better definition of PICS may be warranted to improve the quality of studies regarding PICS. More studies are needed to better understand post-stroke-related pain and fatigue and which therapeutic intervention is best for these patients to optimize daily functioning and quality of life. It is challenging to assess and treat cognition in these patients not knowing how to differentiate structural brain damage from reversible damage. SAH patients should be screened early for complaints of physical, cognitive, and psychological functioning. A multidisciplinary team approach could be beneficial for individual patients if complaints are recognized and if treatment can be initiated in an early phase. More awareness and education in patients and caregivers as well as professionals who are confronted with post-stroke SAH patients is needed.

## Conclusions

A high prevalence of PICS symptoms in patients suffering from aSAH was found four months after ICU discharge. Also, patients with assumed good clinical outcomes showed a high prevalence of PICS symptoms. Future studies on PICS in aSAH patients should include the evaluation of all domains of PICS. Early screening for PICS in aSAH patients may result in a more tailored rehabilitation in these patients.
